# Damage Source Identification of Reinforced Concrete Structure Using Acoustic Emission Technique

**DOI:** 10.1155/2013/870585

**Published:** 2013-08-13

**Authors:** Alireza Panjsetooni, Norazura Muhamad Bunnori, Amir Hossein Vakili

**Affiliations:** ^1^Bakhtar Institute of Higher Education, Ilam, Iran; ^2^School of Civil Engineering, Engineering Campus, Universiti Sains Malaysia (USM), Seri Ampangan, Seberang Perai Selatan, 14300 Nibong Tebal, Pulau Pinang, Malaysia

## Abstract

Acoustic emission (AE) technique is one of the nondestructive evaluation (NDE) techniques that have been considered as the prime candidate for structural health and damage monitoring in loaded structures. This technique was employed for investigation process of damage in reinforced concrete (RC) frame specimens. A number of reinforced concrete RC frames were tested under loading cycle and were simultaneously monitored using AE. The AE test data were analyzed using the AE source location analysis method. The results showed that AE technique is suitable to identify the sources location of damage in RC structures.

## 1. Introduction

The reinforced concrete structures require periodic inspections to ensure the structural safe service. A diagnostic inspection on the current state of the deterioration is necessary for maintenance. In addition, any decisions to repair or retrofit and reconstruct existing structures require real time data. Thus, there is a growing need to evaluate damage and predict remaining capacity life. There are several nondestructive techniques for structure assessment but these testing methods for concrete and steel structures do not provide the full information about the severity of defects in real time. Therefore, there is need for developing an effective nondestructive test method and corresponding evaluation criteria to evaluate their damage level and remaining load capacity before making such decision.

AE technique is a powerful testing tool for real time examination of the behavior of materials deforming under stress [1]. For decades, this technique is being used as the prime candidate for structural health and damage monitoring in loaded structures [2]. This technique has proved to be highly effective especially to assess and measure the damage phenomena taking place inside a structure subjected to mechanical loading [3]. Extensive acoustic emission (AE) studies of RC structures have been reported, and this method was proposed for monitoring of RC structure but more study is needed to develop methods of analyzing the data recorded during the monitoring.

Acoustic emission (AE) is defined as the class of phenomena whereby transient elastic waves are generated by rapid released of energy from localized sources within a material or the transient waves generated [4]. Load conditions that exist in structure have been known to cause materials like steel and concrete to emit AE energy in the form of elastic waves due to various material-relevant damage mechanisms. A developing flaw in these materials emits bursts of AE energy in the form of high frequency sound waves, which propagate within the material and are received by sensors [1]. 

The main objective of this current study was damage evaluation assessment of RC structure with AE source location analysis. Commonly, previous works have been focused on local evaluation of RC beams. However in this research, suitability of this method for global evaluation of RC frame was investigated.

## 2. Methodology

### 2.1. Source Location

One of the most useful applications of AE is in the location of active defects. The location of active defects is calculated by means of the differences between arrival times of a signal at two or more transducers. Linear flaw location is calculated as the following:

(1) $$\begin{array}{c} X=\frac{v\left(t_{a}-t_{b}\right)}{2}, \end{array}$$

where *X* is the distance between the source to the midpoint between two transducers, *t*_*a*_ is the arrival time at transducer *a*, *t*_*b*_ is the time of arrival at transducer *b*, and *v* is a constant determined from the speed of wave propagation through the material [5].

The first step in quantitative AE analysis is the estimation of spatial and temporal parameters of the stress wave source. The estimation of spatial and temporal parameters of the stress wave source is the first step in quantitative AE analysis.

The source location in AE technique is done by measuring the time difference in the arrival at time of an AE signal at different sensors [6]. Generally, the p-wave arrival times are used because they represent the first undisturbed arrival of a stress wave and the easiest to deduce. If at least four sensors detect a discrete stress wave signal, it can be identified as an AE event, and temporal parameters of the stress wave source can be estimated [7]. 

The primary sources of AE are deformation processes such as crack growth and plastic deformation. The AE sources generate and propagate elastic waves in materials in all directions. Ultimately, the elastic waves reach the surface of the material and are detected by sensors attached to the surface of the specimen. AE energy is the total elastic energy released by an AE event occurred at a source [8]. AE energy is defined as follows [9]:

(2) $$\begin{array}{c} E=\int_{t_{0}}^{t_{1}}V_{i}^{2}\left(t\right)dt, \end{array}$$

where *V*_*i*_ is the voltage transient of an *i*th channel, *t*_0_ is the starting time of the voltage transient record, and *t*_1_ is the ending time of the voltage transient record.

## 3. Experimental Procedure

### 3.1. Material Details

A series of experiments was conducted on reinforced concrete (RC) frame. A total of five RC frame specimens were built. The dimensions of RC frames were in length of 2000 mm, height of 1000 mm, and crocs section of 250 × 250 mm. The water to cement ratio was 0.5, and the material proportions were 1 : 3 : 4 : 0.6 by weight of cement, sand, aggregate, and water, respectively. The average compressive strength of concrete at 28 days was 240 Mpa.

### 3.2. Test Monitoring Using AE Technique

**Figure 1:**
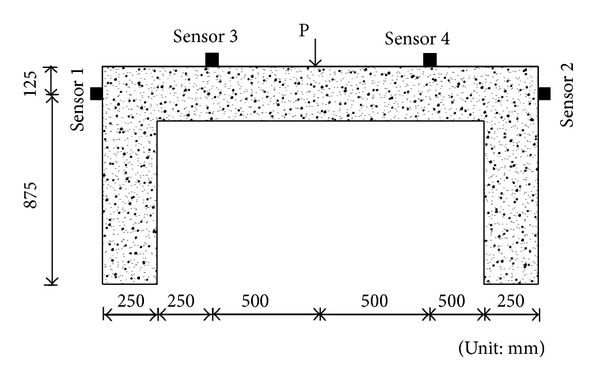
Sensor arrangements for the three-point bending test.

A total of five RC frame specimens described earlier were tested under loading cycle. In order to perform acoustic emission monitoring, an eight-channel AE system (DISP-8PCI) manufactured by Physical Acoustics Corporation (PAC) was employed. Four R6I sensors with the resonance frequency of approximately 60 kHz were used.  Figure 1 shows sensor arrangements for the three-point bending test. The AE systems hardware was set up as threshold level of 45 dB for all channels in order to avoid the possibility of noise effect. The cyclic load pattern was determined. The load was applied in 10 kN steps at midspan of RC frame. The load was applied from 0.5 kN to maximum of each loading cycle (10 kN increment) and held constant for one minute. Then, the load was unloaded from maximum of each loading cycle to 0.5 kN and was held for 2 minutes. The test was monitored by AE throughout the test. The measurements of load, midspan deflection, and AE data were recorded continuously during the three-point bending test.

## 4. Results Analysis and Discussion

**Figure 2:**
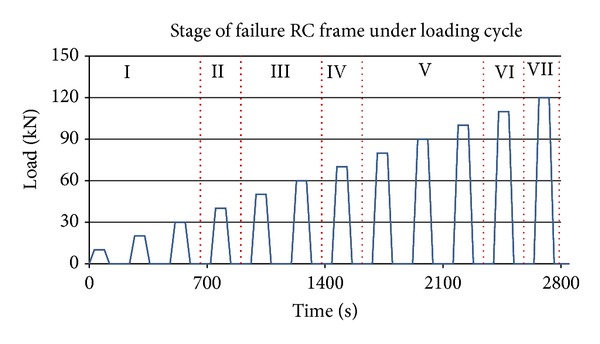
A typical cracks development in the RC frames specimen.

The RC frames described early were tested under loading cycle.  Figure 2 shows typical cracks development in the RC frames specimen. The behaviour of all RC frames under loading cycle can be divided into seven stages of failure namely, (I) micro-cracking at the midspan of RC frame, (II) first flexural cracks at midspan of RC frame, (III) distributed flexural cracks at the midspan of RC frame, (IV) first cracks at the beam-column connection zones, (V) distributed cracks at beam-column connection zones, (VI) damage localization at the beam-column connection zone, (VII) failure at beam-column connection zone.

**Figure 3:**
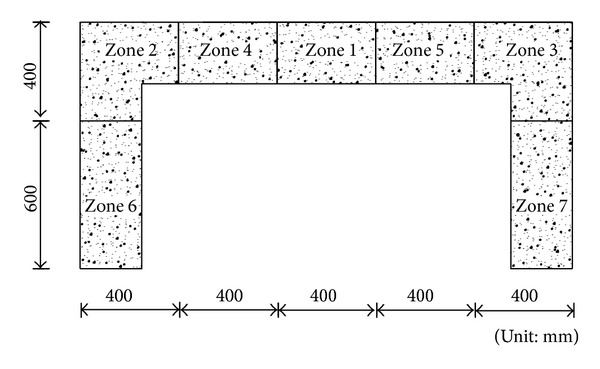
Detail and dimension of specimen zones.

In order to explain AE source location analysis and the behaviour of RC frame specimens under loading cycle, the RC frame specimens were divided into 7 zones which are shown in Figure 3. Zone 1 is associated with the loading zone. Zones 2 and 3 are beam-column connection zone. Zones 6 and 7 are beam-supported region. The AE source location analysis in terms of absolute energy was performed that a sample will be explained.

**Figure 4:**
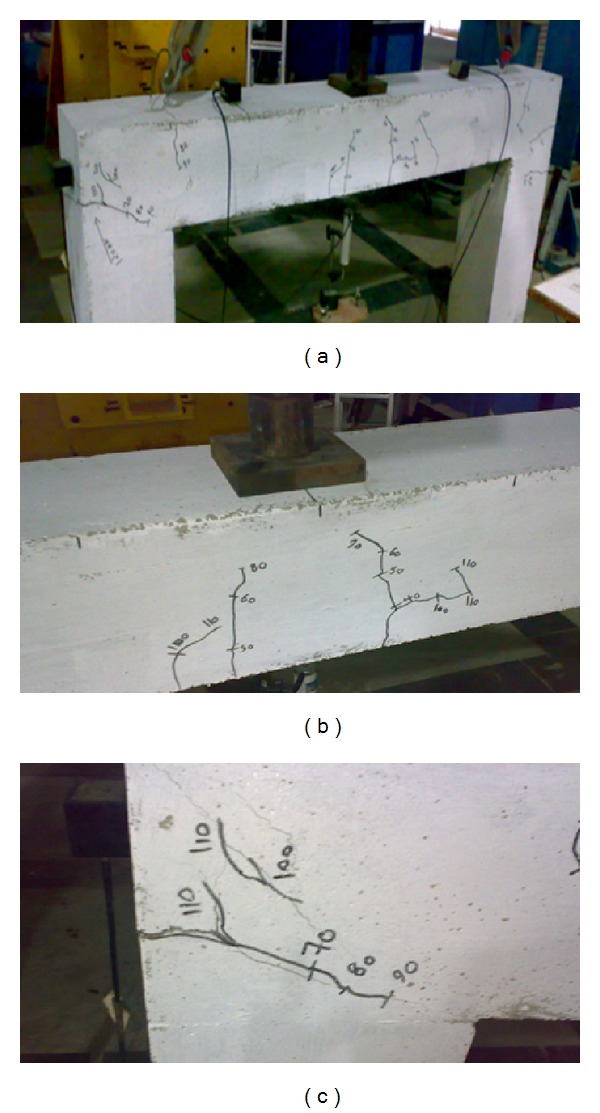
Progression of growth cracking in an RC frame sample—SPRCF1.

*SPRCF1* was tested under loading cycle. The load was applied under twenty load cycles in 10 kN step. This specimen failed at 120 kN, and location of failure was beam-column connection zone.  Figure 4(a) shows a photograph of crack development in SPRCF1. Also, two photographs of crack development in midspan and beam-column connection of this sample are shown in Figures 4(b) and 4(c).

The photographs show that the first cracks are visible in loading cycle 4 (40 kN), and location is at midspan of beam; also, the first cracks which are visible in beam-column are in loading cycle 7 (70 kN). In addition, the photographs show the location of failure in loading cycle 12 (120 kN). A summary of observation and absolute energy versus *X* position during loading cycle is presented in Table 1. The AE source location analysis in terms of absolute energy according to seven stages of failure of the RC frame specimen will be explained and discussed.

**Figure 5:**
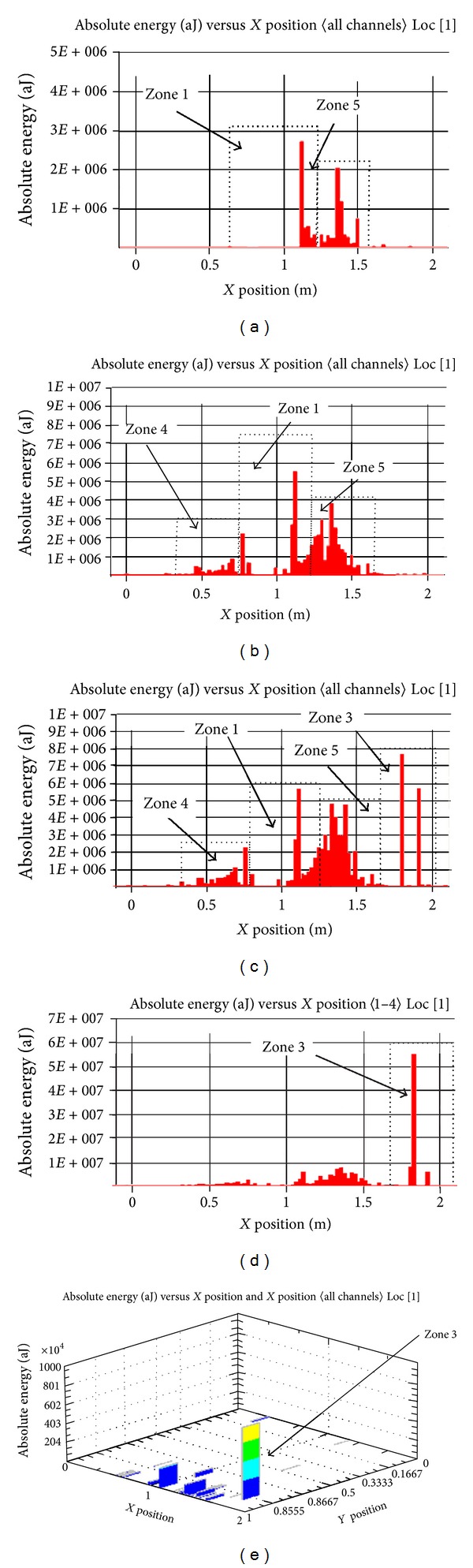
Source location of total emission—SPRCF1.

*In the first stage of failure*, any crack was not visible by the naked eye. During loading cycles 1 to 2, AE activity was in low level throughout the RC frame specimen. Also, during cycle 3, two peaks of absolute energy were observed at zones 4 and 5. These peaks were 2.8 × 10^6^ aJ and 2.1 × 10^6^ aJ at zone 1 and 5, respectively.  Figure 5(a) shows the linear source location in terms of absolute energy and *X* position during cycle 3.

*In the second stage of failure*, first visible cracks were observed at zones 1, 4, and 5.  Figure 5(b) shows the linear source location in terms of absolute energy and *X*-position during cycle 4. The AE were distributed throughout the RC frame specimen with moderate level. The cracks were accompanied by an increase in absolute energy level at zones 1, 4, and 5. However, the highest peaks were estimated, 5.5 × 10^6^, 2.0 × 10^6^, and 3.8 × 10^6^ aJ in zones 1, 4, and 5 respectively.

*In the third stage of failure* cracks were distributed in zones 1, 4, and 5; in this stage, any change was not observed in the highest peaks than that of previous stage. However, the highest peaks were estimated as 5.5 × 10^6^, 2.0 × 10^6^, and 3.8 × 10^6^ aJ in zones 1, 4, and 5, respectively.

*In the fourth stage of failure*, first visible cracks were observed in zone 3 (beam-column connection), and these cracks were accompanied by a high-absolute energy level in this zone.  Figure 5(c) shows the linear source location in terms of absolute energy and *X* position during cycle 7. The cracks were accompanied by an increase in absolute energy level at zone 3 and in this stage, any change was not observed in the highest peaks at zones 1, 4, and 5 than, previous stage. However, the highest peaks were estimated, 5.5 × 10^6^, 2.0 × 10^6^, 3.8 × 10^6^,  2.9 × 10^6^, and 7.8 × 10^6^ aJ in zones 1, 4, 5, and 3, respectively.

*In the sixth stage of failure*, cracks were distributed in zones 1, 4, 5, and 3, any change was not observed in the highest peaks than that of previous stage.

*In the seventh stage of failure*, RC frame failed in zone 3. This phenomenon was only accompanied by an increase in peak of absolute energy at zone 3 from 7.8 × 10^6^ to 5.5 × 10^7^ aJ.  Figure 5(d) shows the linear source location in terms of absolute energy and *X* position, and Figure 5(e) shows the three dimensions of source location in terms of absolute energy during cycle 12 and in the seven stages of failure.

From results obtained of AE source location in stage from microcracks, it has been shown that AE method can be used to obtain the growth of internal microcracks at critical location. Also, the results show that first visible cracks at each location of RC frame specimen increased in cumulative absolute energy in those locations.

In addition, during distributed cracks, cumulative absolute energy was approximately constant. Furthermore, source location of damage can be identified by a comparison between visual observations of the cracks possess and AE source location.

## 5. Conclusions

This paper provides the results from tests on welded steel beam specimen under loading cycle and was monitored by AE throughout the test. On the basis of AE activities and the analysis of signal characteristics using AE source location analysis, the conclusions are presented in the following:

1. The results showed that that AE technique is strongly sensitive with cracks growth in RC frame specimens.
2. The results indicated that AE technique can be used to identify the sources of damage in concrete structure.
3. Results showed that AE can be considered as a viable method to predict the remaining service life of reinforced concrete structure.

## Figures and Tables

**Table 1:** A summary of AE source location analysis.

Cycle no.	Load (kN)	Visual observation	Absolute energy (aJ) [10^6^]			
			Zone 1	Zone 3	Zone 4	Zone 5
1	10	Any cracks were not visible by the naked eye	Not significant			
2	20	Any cracks were not visible by the naked eye	Not significant			
3	30	First cracks at zone 1 and 5 were observed	2.8	—	—	2.0
4	40	The cracks were distributed in zone 1 and 5	2.9	—	—	3.5
5	50	The cracks were distributed in zones 1, 4, and 5	5.5	—	—	3.5
6	60	The cracks were distributed in zones 1, 4, and 5	5.5	—	2.0	3.5
7	70	The first cracks at zone 3 were observed	5.5	7.5	2.0	3.5
8	80	The cracks were distributed in zone 3	5.5	7.8	2.0	3.5
9	90	The cracks were distributed in zone 3	5.5	7.9	2.0	3.5
10	100	The cracks were distributed in zone 3	5.5	8.0	.0	3.5
11	110	The cracks were localized at zone 3	5.5	55	2.0	10
12	120	The RC frame failed at zone 3	5.5	55	2.0	20

## References

[1] Nair A, Cai CS (2010). Acoustic emission monitoring of bridges: review and case studies. *Engineering Structures*.

[2] Surgeon M, Wevers M (1999). Modal analysis of acoustic emission signals from CFRP laminates. *NDT and E International*.

[3] Carpinteri A, Lacidogna G, Niccolini G (2006). Critical behaviour in concrete structures and damage localization by acoustic emission. *Key Engineering Materials*.

[4] Astm E (2006). *Standard Test Method for Measurement of Fracture Toughness. Annual Book of Astm Standards*.

[5] Lozev MG (1997). *Acoustic Emission Monitoring of Steel Bridge Members*.

[6] Shield CK (1997). Comparison of acoustic emission activity in reinforced and prestressed concrete beams under bending. *Construction and Building Materials*.

[7] Schumacher T, Straub D, Higgins C (2012). Toward a probabilistic acoustic emission source location algorithm: a Bayesian approach. *Journal of Sound and Vibration*.

[8] Beattie AG, Jaramillo RA (1974). The measurement of energy in acoustic emission. *Review of Scientific Instruments*.

[9] Miller RK, McIntre P (1987). *Nondestructive Testing Handbook: Volume 5 Acoustic Emission Testing*.

